# Choosing and Doing wisely: triage level I resuscitation a possible new field for starting palliative care and avoiding low-value care – a nationwide matched-pair retrospective cohort study in Taiwan

**DOI:** 10.1186/s12904-020-00590-5

**Published:** 2020-06-20

**Authors:** Chih-Yuan Lin, Yue-Chune Lee

**Affiliations:** 1 Department of Neurology, Taipei City Hospital, Taipei, Taiwan; 2 Institute of Health and Welfare Policy, School of Medicine, National Yang-Ming University, Taipei, Taiwan; 3 Department of Health Care Management, National Taipei University of Nursing and Health, Taipei, Taiwan; 4 Master Program on Trans-disciplinary Long-Term Care and Management, National Yang-Ming University, Taipei, Taiwan

**Keywords:** Emergency care, Triage, Resuscitation, Palliative care, Life-sustaining treatment, Advance care planning, Advanced decision

## Abstract

**Background:**

The association between palliative care and life-sustaining treatment following emergency department (ED) resuscitation is unclear. This study aims to analyze the usage of palliative care and life-sustaining treatments among ED triage level I resuscitation patients based on a nationally representative sample of patients in Taiwan.

**Methods:**

A matched-pair retrospective cohort study was conducted to examine the association between palliative care and outcome variables using multivariate logistic regression and Kaplan–Meier survival analyses. Between 2009 and 2013, 336 ED triage level I resuscitation patients received palliative care services (palliative care group) under a universal health insurance scheme. Retrospective cohort matching was performed with those who received standard care at a ratio of 1:4 (usual care group). Outcome variables included the number of visits to emergency and outpatient departments, hospitalization duration, total medical expenses, utilization of life-sustaining treatments, and duration of survival following ED triage level I resuscitation.

**Results:**

The mean survival duration following level I resuscitation was less than 1 year. Palliative care was administered to 15% of the resuscitation cohort. The palliative care group received significantly less life-sustaining treatment than did the usual care group.

**Conclusion:**

Among patients who underwent level I resuscitation, palliative care was inversely correlated with the scope of life-sustaining treatments. Furthermore, triage level I resuscitation status may present a possible new field for starting palliative care intervention and reducing low-value care.

## Background

Emergency department (ED) resuscitation refers to a critical time-sensitive state requiring immediate resuscitation [1, 2]. The fundamental task of critical care is to initiate timely stabilization, diagnosis, and therapeutic interventions to save a patient’s life and improve their prognosis. The constant updating of evidence-based resuscitation, advanced life support, and rapid development of organ replacement life-sustaining treatments have led to better progress in resuscitation practices. However, the inevitability of end-of-life situations leads to complex situations and difficulties in care decision making [3]. Triage level I resuscitation accounts for about 1.9 ~ 5% of all ED visits [4–7]. In previous studies conducted in Asia, the overall out-of-hospital cardiac arrest survival rates upon hospital discharge have ranged from 0.5 to 8.5% [8, 9]. The post-cardiac arrest resuscitation mean one-year survival rate was shown to be approximately 5% [10]. Many survivors experience neurological impairment [9] and further comorbidities can lead to long-term disability or initiate an end-of-life state, particularly for frail patients [11–14].

Early identification of the end-of-life state is key to avoiding low-value emergency care [15]. Medical service providers must make important decisions pertaining to resuscitation [16–18]. The Choosing Wisely Campaign (American College of Emergency Physicians) recommends that decision-makers begin palliative and hospice care in the ED [19]. The second set of the Choosing Wisely campaign also recommended that medical service providers engage with patients’ shared decision making [20]. However, that a large number of patients who undergo triage level I resuscitation frequently miss opportunities for considering palliative care [13].

In January 2019, the Taiwan Ministry of Health and Welfare (MoHW) launched the Patient Autonomy Act, aimed at ensuring that all adults or surrogate(s) have access to counseling related to care options, including refusal of life-sustaining treatment for terminal diseases, irreversible comatose condition, vegetative state, advanced dementia, and other intractable illnesses or incurable diseases [21]. Medical decisions must account for clinical scenarios, previous experience making medical decisions, past experiences related to death and dying [22], and the social situation involving friends and family [23]. However, evidence-based post-resuscitation data for family meetings and discussion is still lacking. Therefore, this study compared resuscitation patients who received palliative care with those who received usual care in terms of medical utilization, medical expenses, and the provision of life-sustaining treatment. Our findings provide evidence that may extend palliative care to meet patients’ need and reduce low-value care.

## Methods

### Setting

The National Health Insurance (NHI) program in Taiwan has provided hospice care since the first hospital-based hospice ward was opened in 1990, and palliative care since the Hospice Palliative Care Act was passed in 2000. The care settings include inpatient hospice wards and home and community hospice care. Palliative services were initially intended for patients with cancer or motor neuron diseases; however, it was expanded in 2009 to include eight categories of non-cancer patients [24]. Since 2006, NHI IC cards have included a statement indicating a desire to receive palliative care as opposed to life-sustaining treatment. As of 2015, approximately 15% of cardholders signed documents expressing their willingness to undergo palliative care [25]. In 2015, roughly 37,000 patients with a terminal illness received palliative care [25].

### Study design and data source

This nationwide retrospective cohort study was based on the National Health Insurance Research Database (NHIRD) of Taiwan. We obtained NHIRD data from a sample of roughly one million nationally representative claims for inpatient and ambulatory care between September 1, 2009, to December 31, 2013 [26, 27]. The claim data included the date of ED; disease diagnoses based on the International Classification of Disease, Ninth Revision, Clinical Modification codes, laboratory workup, and medication. NHIRD was validated for accuracy in diagnostic coding [28], comorbidities [29], severity [30], and end-of-life state healthcare resource utilization [31].

### Ethics

In accordance with regulations of the National Health Research Institutes, patient identification information was anonymized, such that informed consent was not required. This study was approved by the Institutional Review Board of Yang-Ming University (YM-107035E). No funding was received to support this research.

### Identification of study cohort

Palliative care was extended to non-cancer patients in 2009; therefore, this study focused on the period between September 1, 2009, and December 31, 2013. Inclusion criteria included ED triage level I resuscitation during the study period. The palliative care sub-group included patients who received palliative care after post-triage level I resuscitation. Patients who received palliative care prior to the date of ED resuscitation were excluded. The date of the first palliative service was adopted as the index date. Patients in the palliative care subgroup were matched at a ratio of 1:4 with patients who received usual care in terms of event year, season, gender, age, and Charlson Comorbidity Index (CCI). Analysis was performed on the utilization of medical services from the index date until the patient died or the end of the study period. Cases were evaluated in terms of patient disposition, medical utilization, and medical outcomes.

### Variables

The selection of outcome and control variables was based on a conceptual framework describing interventions specific to life-sustaining treatment [32]. The sociodemographic characteristics considered in this analysis included gender, age, occupation, and living area. The CCI was used to identify disease characteristics 1 year prior to the index date. Outcome variables included the number of visits to EDs and outpatient departments, the duration of hospitalization (in days), duration of survival after the index date (in days), total medical expenses, and life-sustaining treatments (including cardiopulmonary resuscitation, extracorporeal membrane oxygenation, hemodialysis, intensive unit care, intraaortic balloon pumping; mechanical ventilation, and nasogastric tube usage). These outcomes were derived from codes listed on inpatient claims.

### Statistical methods

Baseline patient characteristics, medical utilization, and outcomes were compared between (1) the palliative group, and (2) usual care group. Differences in outcome variables were examined across gender, age, survival status, occupation, income, and CCI. ICD-9 codes for principal diagnoses, including palliative services-related codes from inpatient and outpatient NHI claims in the year prior to death, were used to identify comorbidities. The Deyo-Charlson comorbidity index was calculated using ICD-9 codes and categorized as 0, 1, or ≧2 comorbid conditions. Survival duration was based on the interval between the date of ED resuscitation and the date of death or research termination. Continuous variables and categorical variables were compared between groups using t-tests and the Chi-squared test, respectively. The distribution of covariates between groups was also assessed using Chi-square tests and t-tests, where a p-value of < 0.05 indicated a difference of statistical significance. Data with a skewed continuous distribution was described using the median and 25th and 75th percentiles (interquartile range). Kaplan–Meier survival analysis was used to analyze between-group differences related to survival, medical expenses, and the usage of life-sustaining treatments, in conjunction with the log-rank test to determine statistical significance. To obtain the contribution of each predictor to the overall explanatory power of the model, we conducted further subgroup analysis for each stratum of gender, age group, income, living area, CCI and occupation of palliative service and life-sustaining treatment using the full model without stratifying Multivariate logistic regression was used with one dichotomous dependent variable; i.e., whether or not life-sustaining treatment was implemented. Predictor variables included gender, age, income, living area, and CCI. Adjusted odds ratios with 95% confidence intervals were derived from logistic regression analysis, with statistical significance set at a p-value < 0.05 following adjustment for risk factors. All analysis was performed using Statistical Analysis Software version 9.4 (SAS Institute Inc., Cary, NC, USA).

## Results

### Baseline characteristics

**Fig. 1:**
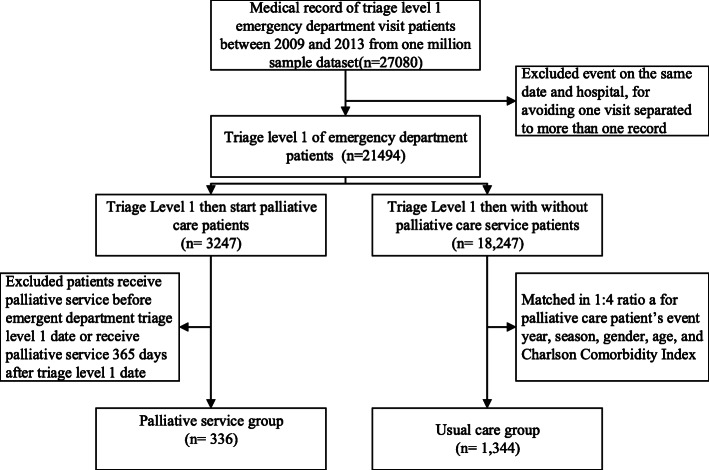
Patient flow

During this 5-year study period, we identified 21,494 cases of ED triage level I resuscitation, which included 336 subjects who received palliative services subsequent to resuscitation. In accordance with the Pitman efficiency index [33], those cases were matched with controls at a ratio of 1:4 (*n* = 1344), resulting in a study cohort of 1680 resuscitation cases (Fig. 1). As shown in Fig. 1, the patients who received palliative care made up 15% of the entire ED triage level I resuscitation cohort. There were no significant differences between cases and controls in terms of sociodemographic variables, including gender, age, income, occupation, place of residence, or comorbidities (see Table 1). In the study cohort, the mean age was 70 years and 61.3% were male. The median insured salary was 880 USD per month, and more than 93% were CCI > 1. Most of the covariates remained comparable between the two groups throughout the follow-up period. Principal ED diagnoses were aggregated using single-level clinical classification software developed by the Agency for Healthcare Research [34]. ED discharge classifications in the palliative group were cancer-related diagnoses and organ-specific high impact time-sensitive conditions. In contrast, those in the usual care group included a variety of residual codes and organ-specific high impact time-sensitive conditions (Supplementary Table 1).

**Table 1:** Baseline demographics of all eligibles among the resuscitation patients with palliative care and usual care

	Before Match				*P*-value	Post Match				*P*-value
	Palliative		Usual care			Palliative		Usual care		
	(*n* = 346)		(*n* = 18,615)			(*n* = 336)		(*n* = 1344)		
	*n*	(%)	*n*	(%)		*n*	(%)	*n*	(%)	
Sex										
Female	136	39.3	8081	43.4	0.13	130	38.7	520	38.7	0.99
Male	210	60.7	10,534	56.6		206	61.3	824	61.3	
Age										
18–64	119	34.4	8465	45.5	<.0001	110	32.7	448	33.3	0.84
≥ 65	227	65.6	10,150	54.5		226	67.3	896	66.7	
Charlson Comorbidity Index										
CCI = 0	5	1.4	5464	29.3	<.0001	5	1.5	26	1.9	0.68
CCI = 1	14	4.1	3363	18.1		14	4.2	68	5.1	
CCI > 1	327	94.5	9788	52.6		317	94.3	1250	93	
Income level										
Quintile 1	144	41.7	8408	45.2	0.269	140	41.7	614	45.7	0.11
Quintile 2	104	30.1	5587	30.0		100	29.8	419	31.2	
Quintile 3	98	28.2	4620	24.8		96	28.5	311	23.1	
Occupation										
Dependents of the insured individuals	134	38.7	6031	32.4	0.0006	132	39.3	493	36.7	0.92
Civil servants, teachers, military personnel, veterans	37	10.7	1760	9.5		37	11.0	152	11.3	
Nonmanual workers and professionals	14	4.1	2065	11.1		13	3.9	52	3.9	
Manual workers	115	33.2	6132	33.0		111	33.0	476	35.4	
Other	46	13.3	2540	13.6		43	12.8	168	12.5	
Missing	0	0.0	87	0.4		0	0.0	3	0.2	
Urbanization										
Urban	85	24.6	4735	25.4	0.1737	83	24.7	309	23.0	0.29
Suburban	108	31.2	5077	27.3		104	31.0	365	27.2	
Rural	153	44.2	8666	46.6		149	44.3	667	49.6	
Missing	0	0.0	137	0.7		0	0.0	3	0.2	

### Medical expenses and utilization of life-sustaining treatment

Table 2 lists data pertaining to duration of survival, medical expenses, and utilization of life-sustaining treatment in the palliative group and usual care group. The mean survival durations after resuscitation were as follows: palliative group (107.3 days) and usual care group (302.2 days). Compared with the palliative group, the usual care group included 5-fold more ED visits, 3-fold more hospitalizations, and 4-fold more ICU admissions. Compared with the palliative group, the usual care group had significantly higher medical expenses, hemodialysis, cardiopulmonary resuscitation, ventilator use, and life-sustaining treatment. No significant differences were observed between the two groups in terms of nasogastric tube feeding (*p* = 0.28).

**Table 2:** Palliative care and usual care patients: survival, medical utilization and life-sustaining treatment use

	Palliative care		Usual care		*p-value*
	(*n* = 336)		(*n* = 1344)		
	Mean	(SD)	Mean	(SD)	
Post ED resuscitation survival days	107.3	127.20	302.2	406.70	<.0001
Post palliative survival days	48.7	81.87	273.8	384.90	<.0001
ED visit	0.4	1.11	2.1	6.12	<.0001
Hospitalization	0.5	1.36	1.6	3.10	<.0001
ICU admission	0.003	0.055	0.014	0.118	0.011
OPD expense (USD)	256.2	776.1	4477.8	11,980.5	<.0001
Hospitalization expense (USD)	1233.1	5089.0	5390.2	13,118.6	<.0001
All medical expense (USD)	1489.3	5312.0	9898.1	19,572.0	<.0001
LST-Total LSTs usage	9.0	21.20	22.2	62.35	<.0001
LST-NGT feeding	4.9	8.89	4.3	9.98	0.28
LST-IABP	–	–	0.03	0.24	–
LST-ECMO	–	–	0.02	0.20	–
LST-Hemodialysis	1.9	12.61	13.5	56.94	<.0001
LST-CPR	0.1	0.27	0.2	0.46	<.0001
LST-Ventilator	2.1	6.79	4.1	14.58	0.0003

*CPR* Cardiopulmonary resuscitation, *ECMO* Extracorporeal membrane oxygenation, *ED* Emergency department, *IABP* Intraaortic balloon pumping, *ICU* Intensive care unit, *LST* Life-sustaining treatment, *NGT* Nasogastric tube, *OPD* Outpatient department, *USD* US Dollar, convert USD/TWD 1:30

### Life-sustaining treatments: predictors

**Fig. 2:**
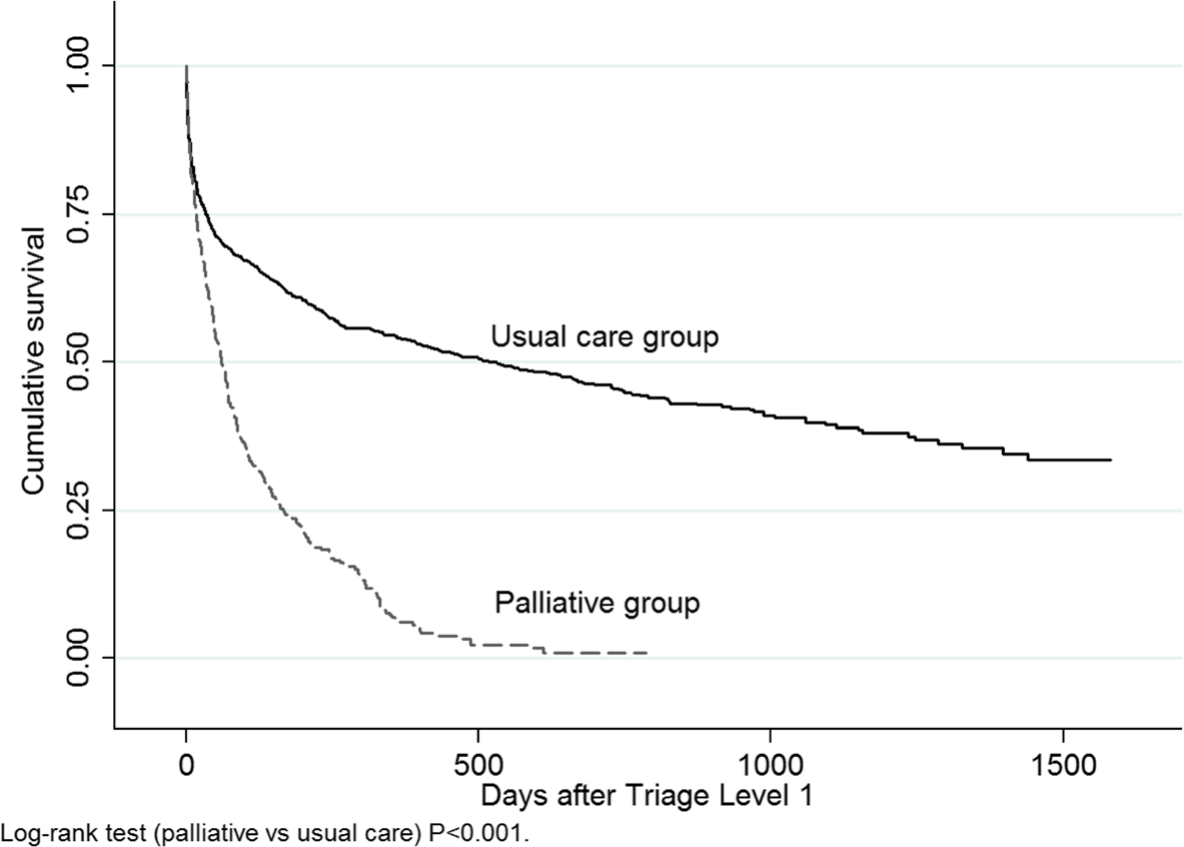
Kaplan-Meier estimate plot for survival days post-emergency department resuscitation

Multivariate analysis was used to examine various clinical factors of life-sustaining treatments administered after the index date (Table 3). The palliative group significantly exceeded the usual care group in terms of using nasogastric tube feeding (adjusted odds ratio = 2.45; 95% CI 1.87–3.20; *p* < .0001). The usual care group significantly exceeded the palliative group in terms of hemodialysis usage (adjusted odds ratio = 0.39; 95% CI 0.25–0.59; *p* < .0001), cardiopulmonary resuscitation usage (adjusted odds ratio = 0.24; 95% CI 0.15–0.38; *p* < .0001), and ventilator usage (adjusted odds ratio = 0.42; 95% CI 0.32–0.54; *p* < .0001). Kaplan–Meier survival curves were significantly lower in the palliative group than in the usual care group (Log-rank test, *p* < 0.001) (Fig. 2).

**Table 3:** Multivariate generalized linear model analysis of life-sustaining treatment

	Nasogastric tube feeding			Hemodialysis			Cardiopulmonary resuscitation			Ventilator		
Variables	aOR (95 CI)		*p*	aOR (95% CI)		*p*	aOR (95% CI)		*p*	aOR (95% CI)		*p*
Intercept	–		0.911	–		0.005	–		0.0001	–		0.469
**Palliative care** (Ref: usual care) Yes	2.45	(1.87–3.20)	<.0001	0.39	(0.25–0.59)	<.0001	0.24	(0.15–0.38)	<.0001	0.42	(0.32–0.54)	<.0001
**Sociodemographic characteristics Gender** (Ref: female) Male	0.94	(0.77–1.16)	0.57	0.61	(0.47–0.81)	0.0004	1.25	(0.96–1.63)	0.091	1.22	(0.99–1.50)	0.062
**Age group** (Ref: 18–65 yrs) ≧65 years old	1.33	(1.06–1.66)	0.014	0.69	(0.51–0.92)	0.016	0.70	(0.50–0.87)	0.003	0.80	(0.64–0.99)	0.049
**Insurance salary** (Ref:<USD 730) USD 730 ~ 920	1.29	(0.93–1.80)	0.134	1.12	(0.73–1.75)	0.566	0.67	(0.44–1.01)	0.053	0.98	(0.71–1.36)	0.900
>USD 920	0.96	(0.73–1.27)	0.788	1.15	(0.80–1.67)	0.287	0.95	(0.68–1.33)	0.760	1.02	(0.78–1.34)	0.900
**Occupation** (Ref: dependents of the insured individuals) Civil servants, teachers, military personnel, veterans	1.30	(0.87–1.93)	0.196	1.25	(0.75–2.06)	0.072	1.30	(0.81–2.08)	0.281	1.42	(0.97–2.09)	0.072
Nonmanual workers and professionals	1.36	(0.79–2.35)	0.273	0.79	(0.37–1.72)	0.627	0.91	(0.48–1.72)	0.773	0.99	(0.58–1.70)	0.967
Manual workers	1.09	(0.83–1.43)	0.533	1.05	(0.73–1.53)	0.330	0.70	(0.68–0.97)	0.033	0.96	(0.73–1.25)	0.748
Other	0.95	(0.64–1.41)	0.803	1.05	(0.63–1.77)	0.474	1.11	(0.68–1.81)	0.682	1.23	(0.83–1.81)	0.307
**Urbanization** (Ref: urban) Suburban	0.83	(0.62–1.09)	0.183	0.65	(0.45–0.93)	0.156	0.93	(0.66–1.32)	0.699	0.77	(0.59–1.02)	0.067
Rural	0.93	(0.71–1.21)	0.579	0.65	(0.44–0.92)	0.022	1.08	(0.78–1.49)	0.662	0.89	(0.69–1.16)	0.391
**Disease characteristics Charlson Comorbidity Index** (Ref: CCI>1) CCI = 1	1.15	(0.72–1.82)	0.569	< 0.001	(0.01–999.99)	0.977	1.20	(0.69–2.05)	0.530	1.72	(1.08–2.74)	0.022
CCI = 0	0.80	(0.38–1.69)	0.550	0.17	(0.05–0.56)	0.003	1.73	(0.77–3.86)	0.185	1.49	(0.70–3.19)	0.303

*aOR* Adjusted odds ratio, *P p-*value, *CI* Confidence interval, *USD* US dollar

## Discussion

Our results indicated that opting for palliative care was correlated with a shorter duration of life with disability, medical expenses, and life-sustaining treatment. Survival duration in the palliative group was significantly shorter than in the usual care group. Nonetheless, both groups qualified for end-of-life services (i.e., survival of less than 1 year) [35]. Note that patients in the palliative group made fewer visits to ED and outpatient departments, and were less likely to be hospitalized. Moreover, also that patients in the palliative group underwent fewer life-sustaining treatments and incurred fewer medical expenses.

### Methods discussion

This study compared patients who did or did not undergo palliative care after successful ED resuscitation. Note that this methodology could be confounded by the indication for palliative services. Since 2009, Taiwan’s NHI palliative care coverage expanded to eight non-cancer irreversible advanced stage organ-system dysfunctions [24]. The NHI is tasked with providing comprehensive, low-cost healthcare [36] and high-quality palliative care [37]. Taiwan expanded palliative care policy to reimburse full palliative care services, which increased access to cancer [38] and non-cancer palliative care in Taiwan [39]. Meanwhile, decisions pertaining to palliative care are affected by early palliative care consultation services [40], provider reimbursement policy [41], patient awareness of prognosis and completion of advances directives [42], and family awareness of diagnosis and prognosis [40]. It appears that healthcare utilization is determined largely by the fact that patients in the usual care group far outlived those in the palliative group post-resuscitation. The greater use of medical utilization and life-sustaining treatment is most likely due to the longer post-resuscitation survival in the usual care group. Compared to the usual care group, the palliative group had a shorter survival duration and less extensive utilization of life-sustaining treatments. Our primary objective in this paper was to promote conversation pertaining to palliative care between patients and medical practitioners in order to identify suitable candidates for palliative care and thereby prevent unnecessary suffering.

### Results discussion

Contemporary palliative care practice and research are disease-oriented (cancer and non-cancer disease) [43, 44] and setting-oriented (palliative ward, ED, intensive care unit, home-based, long-term care facility, and community-based setting) approaches [45–48]. ED level I resuscitation is a common pathway of patients with advanced and progressive deterioration illnesses, as well as those with incurable frailty or co-existing conditions. In this study, the post-ED resuscitation survival duration of all patients (both groups) was 351 days, which clearly falls within the definition of palliative care [35]. In cases where the patient is in an incurable or severely disabled state post-resuscitation, palliative care should be considered. It has been estimated that total health expenditures could be reduced by 30% simply by avoiding repeated visits to the ED or unnecessary ICU hospitalization during the final months of life [49–51]. Our results indicate that decisions pertaining to palliative care could be considered earlier to reduce suffering and decrease expenses for low-value medical care [49, 52, 53]. Paradoxically, tube feeding was higher in the palliative care group; however, this has also been reported in previous studies conducted in Asia [54, 55]. Many Asians believe that providing artificial nutrition is necessary to prevent dying patients from becoming what is referred to as ‘hungry ghosts’ [56, 57].

This study has a number of limitations. First, the patients included in the palliative group were not selected at random. Nonetheless, claims data from the NHIRD is representative of critical care in Taiwan with respect to ED size, location, academic affiliation, and patient characteristics. Second, we were unable to determine the reasons underlying the assignment of participants to palliative care. Moreover, we were unable to measure the number of palliative care patients who received life-sustaining treatment or the corresponding timelines. Third, this study did not have access to data related to sociocultural factors, the nature of each case, personal medical experience, or family dynamics.

## Conclusions

The implementation of palliative care was correlated with fewer life-sustaining treatments following resuscitation. This study provides real-world data pertaining to post-resuscitation care, which could be of value to clinicians, patients, and their families undergoing with evidence-based counseling when facing life-sustaining treatment share decision making. We believe that ED triage level I resuscitation status is a potential starting point for early palliative case finding and end-of-life counseling.

## Supplementary information

**Additional file 1: Supplementary Table 1.** Top 20 categories of single-level clinical classifications: Palliative and usual care groups

## Data Availability

The data that support the findings of this study are available from Taiwan National Health Insurance Research Database but restrictions apply to the availability of these data, which were used under license for the current study, and so are not publicly available. Data are however available from the academic request and with permission of Taiwan National Health Insurance Administration.

## Abbreviations

CCI: Charlson Comorbidity Index
CPR: Cardiopulmonary resuscitation
ECMO: Extracorporeal membrane oxygenation
ED: Emergency department
IABP: Intraaortic balloon pumping
ICU: Intensive care unit
MoHW: Ministry of Health and Welfare
NHI: National Health Insurance
NGT: Nasogastric tube
NHIRD: National Health Insurance Research Database
OPD: Outpatient department
USD: US Dollar
