# Supplementary material for: Choosing and Doing wisely: triage level I resuscitation a possible new field for starting palliative care and avoiding low-value care – a nationwide matched-pair retrospective cohort study in Taiwan
Source: BMC Palliat Care. 2020 Jun 20;19:87. doi: 10.1186/s12904-020-00590-5 (PMC7305586; doi:10.1186/s12904-020-00590-5)
Supplement: Supplementary file 1 — Additional file 1: Supplementary Table 1. Top 20 categories of single-level clinical classifications: Palliative and usual care groups [file 12904_2020_590_MOESM1_ESM.docx]

**Supplementary Table 1** Top 20 categories of single-level clinical classifications: palliative and usual care groups

| **No** | **Palliative group** | **n** | **(%)** | **Usual care group** | **n** | **(%)** |
| --- | --- | --- | --- | --- | --- | --- |
| **1** | **Cancer of bronchus; lung** | **31** | **9.23** | **Residual codes; unclassified** | **139** | **10.34** |
| **2** | **Pneumonia (except TB)** | **26** | **7.74** | **Respiratory failure (adult)** | **83** | **6.18** |
| **3** | **Lower respiratory disease** | **20** | **5.95** | **Cardiac arrest & VT** | **79** | **5.88** |
| **4** | **Cancer of head and neck** | **18** | **5.36** | **Pneumonia (except TB)** | **75** | **5.58** |
| **5** | **Respiratory failure (adult)** | **17** | **5.06** | **Acute CVA** | **70** | **5.21** |
| **6** | **Cancer of liver intrahepatic** | **13** | **3.87** | **Lower respiratory disease** | **66** | **4.91** |
| **7** | **Acute CVA** | **12** | **3.57** | **Chronic kidney disease** | **63** | **4.69** |
| **8** | **Coma; stupor; brain damage** | **11** | **3.27** | **Coma; stupor; brain damage** | **60** | **4.46** |
| **9** | **Fever of unknown origin** | **11** | **3.27** | **Other endocrine disorders** | **53** | **3.94** |
| **10** | **Cancer of rectum and anus** | **10** | **2.98** | **Gastrointestinal hemorrhage** | **30** | **2.23** |
| **11** | **Residual codes; unclassified** | **9** | **2.68** | **Cancer of bronchus; lung** | **29** | **2.16** |
| **12** | **Other endocrine disorders** | **9** | **2.68** | **Septicemia (except in labor)** | **29** | **2.16** |
| **13** | **Shock** | **8** | **2.38** | **Shock** | **28** | **2.08** |
| **14** | **Gastrointestinal hemorrhage** | **7** | **2.08** | **Other liver diseases** | **26** | **1.93** |
| **15** | **Septicemia (except in labor)** | **7** | **2.08** | **COPD and bronchiectasis** | **26** | **1.93** |
| **16** | **Cancer of colon** | **6** | **1.79** | **Fever of unknown origin** | **23** | **1.71** |
| **17** | **E Codes: Place of occurrence** | **5** | **1.49** | **E Codes: Place of occurrence** | **21** | **1.56** |
| **18** | **Epilepsy; convulsions** | **5** | **1.49** | **CHF; nonhypertensive** | **21** | **1.56** |
| **19** | **Cancer; other respiratory** | **5** | **1.49** | **Acute myocardial infarction** | **21** | **1.56** |
| **20** | **Chronic kidney disease** | **4** | **1.19** | **DM with complications** | **21** | **1.56** |
|  | **Total** | **234** | **69.64** |  | **963** | **71.65** |

CHF: congestive heart failure; COPD: chronic obstructive pulmonary disease; CVA: cerebrovascular disease; DM: Diabetes mellitus TB: tuberculosis; VT: ventricular fibrillation
